# Neutrophil Effector Functions Are Not Impaired in Duffy Antigen Receptor for Chemokines (DARC)-Null Black South Africans

**DOI:** 10.3389/fimmu.2019.00551

**Published:** 2019-03-26

**Authors:** Kewreshini K. Naidoo, Ayanda Ngubane, Pedzisai Gaza, Amber Moodley, Thumbi Ndung'u, Christina F. Thobakgale

**Affiliations:** ^1^HIV Pathogenesis Programme, Nelson R. Mandela School of Medicine, Doris Duke Medical Research Institute, University of KwaZulu-Natal, Durban, South Africa; ^2^Females Rising through Education, Support and Health (FRESH), Durban, South Africa; ^3^Africa Health Research Institute (AHRI), Nelson R. Mandela School of Medicine, University of KwaZulu-Natal, Durban, South Africa; ^4^Ragon Institute of Massachusetts General Hospital, Massachusetts Institute of Technology, and Harvard University, Cambridge, MA, United States; ^5^Max Planck Institute for Infection Biology, Berlin, Germany; ^6^School of Pathology, National Institute for Communicable Diseases, University of the Witwatersrand, Johannesburg, South Africa

**Keywords:** neutrophil, function, DARC, duffy, HIV-1

## Abstract

Neutrophils are well-recognized for their pathogen killing mechanisms and disorders of neutrophil count and function are associated with recurrent infections. The Duffy Antigen Receptor for Chemokines (DARC)-null genotype is predominant in sub-Saharan African ancestry populations and is the major genetic determinant of benign ethnic neutropenia which has been associated with increased risk of Human Immunodeficiency Virus (HIV)-1 acquisition and mother-to-child transmission. However, the impact of DARC-null-linked neutropenia on HIV disease progression remains controversial. While the DARC-null genotype is associated with low numbers of circulating neutrophils, the effects of the polymorphism on neutrophil functions is unknown. We investigated the impact of the DARC-null trait and lower absolute neutrophil counts (ANCs) on key neutrophil effector functions [proteolytic activity within the phagosome following Fc receptor-mediated phagocytosis, reactive oxygen species (ROS) production, and neutrophil extracellular trap (NET) formation] in 20 HIV negative and 22 HIV-1 chronically infected black South Africans. Phagosome maturation was measured by flow cytometry following Fc-mediated uptake of IgG opsonized beads; ROS production was measured by chemi-luminescence after activation of neutrophils with phorbol 12-myristate 13-acetate (PMA). Activated neutrophils were also visualized by fluorescent microscopy for NET quantification. Study subjects were genotyped for the DARC trait using TaqMan allelic discrimination assays and ANCs were measured by full blood count. As expected, the DARC-null polymorphism was highly prevalent in our participant cohort (69%) and was strongly associated with lower ANCs in uninfected (*p* = 0.0007) and HIV-1 infected (*p* = 0.03) subjects. We observed enhanced proteolytic activity within the phagosome in the absence of DARC at 10 min (*p* = 0.05 and *p* = 0.009) and 60 min (*p* = 0.05 and *p* = 0.07) in uninfected and HIV-1 infected subjects, respectively. ROS was unaffected by DARC trait irrespective of HIV status. Furthermore, formation of NETs was reduced in neutrophils from DARC-null subjects (*p* = 0.04) following prolonged *in vitro* stimulation, but only in HIV-1 infected subjects. The data indicate differential neutrophil function in the absence of DARC that may be moderately modulated by HIV-1 infection but overall, the data suggest that DARC-null trait is not deleterious to neutrophil effector functions in African populations.

## Introduction

Neutrophils constitute the earliest and most dominant innate immune response to invading pathogens. Their role in microbial clearance such as fungal and extracellular bacterial infections is well-documented (1, 2), and there is evidence of the key role of neutrophils in viral immunity (3–5). Investigations of neutrophil responses in Human Immunodeficiency Virus (HIV)-1 infection have largely focused on susceptibility to bacterial and fungal co-infections. Only recently have the direct interactions between neutrophils and HIV-1 been fully appreciated (6).

Disorders of neutrophil counts (neutropenia) and function are usually associated with recurrent infection complications, particularly in severe cases where the absolute neutrophil count (ANC) drops to below 500 cells/mm^3^ (7, 8). Congenital disorders driven by diminished neutrophil production and release from the bone marrow exhibit a higher risk of microbial infections in comparison to peripheral acquired neutropenia (9). Ethnic neutropenia is the most frequent form of congenital neutropenia globally, most common in persons of African descent. Clinically, the condition is considered moderate and is not associated with obvious increased oral and systemic infection incidence as observed in other forms of neutropenia (7). It is unclear whether ethnic neutropenia is associated with any defects in neutrophil functions.

The Duffy Antigen Receptor for Chemokines (DARC), a glycosylated cell membrane receptor, is encoded by the DARC gene located on chromosome 1. A single nucleotide polymorphism (-46T>C) in the DARC promoter region abolishes gene expression and results in selective loss of DARC expression on erythrocytes (10). This DARC polymorphism has been identified as the principal genetic determinant of ethnic neutropenia (11). However, the mechanism through which the DARC-null polymorphism induces neutropenia remains poorly understood.

Previous studies have established an association between lower circulating neutrophils exhibited in the absence of DARC and increased risk of HIV acquisition and mother-to-child transmission (12–14). Following HIV infection, it has been suggested that the DARC-null state is accompanied with slower disease progression and that the polymorphism may impart a survival advantage in leukopenic HIV infected individuals (12, 15). Conversely, other reports contesting these findings demonstrated that DARC status did not impact the rate of disease progression based on viral load set points, CD4+ T cell decline or progression to AIDS (16–18).

Overall, the evidence appears stronger for an association between DARC-null-linked ethnic neutropenia and HIV acquisition than it does for its role in HIV disease progression. However, it is plausible that the influence of the DARC-null trait is complex, with many of its underlying unknown components contributing to HIV pathogenesis. While the DARC receptor is not expressed on neutrophils, the absence of DARC is a significant factor associated with low circulating neutrophils. It has been suggested that DARC expression regulates circulating chemokine levels that may affect neutrophil chemotaxis, migration and localization. However, the effect of the DARC-null polymorphism on neutrophil effector functions has not been investigated. Here we aimed to determine the influence of the DARC genotype on neutrophil effector functions in HIV-1 negative vs. HIV-1 chronically infected Zulu/Xhosa black individuals in Durban, South Africa. We hypothesized that neutrophil effector mechanisms including phagosome maturation, production of reactive oxygen species (ROS), or formation of neutrophil extracellular traps (NETs) would be impaired in the absence of DARC and that this dysfunction would be more distinct in HIV infected persons compared to uninfected individuals.

## Materials and Methods

### Study Participants

HIV-1 negative participants (*n* = 20) were recruited from the Females Rising through Education, Support and Health (FRESH) cohort, a socioeconomic empowerment programme that longitudinally monitors HIV-1 uninfected high risk women for acute HIV-1 infection (19, 20). HIV-1 subtype C chronically infected treatment naïve participants (*n* = 22) were recruited from the HIV Pathogenesis Programme (HPP) Acute Infection Cohort, in which individuals with acute or recent HIV-1 infection are longitudinally followed (21). In both cohorts, participants are recruited from the Umlazi Township in Durban, KwaZulu-Natal, South Africa. The protocols were approved by the University of KwaZulu-Natal Biomedical Research Ethics Committee and the Partners Human Research Committee. All subjects gave written informed consent in accordance with the Declaration of Helsinki.

### ANCs, CD4 Counts and Viral Loads

Blood for full blood counts, CD4+ T cell count measurement and viral load quantification was collected in EDTA anticoagulant vacutainer tubes [Becton Dickinson (BD), Franklin Lakes, New Jersey, USA]. ANCs were enumerated by full blood count using the automated XN 1000 Hematology Analyzer (Sysmex, Kobe, Hyogo, Japan). CD4 counts were measured using BD Trucount and analyzed on a four-parameter FACS Calibur flow cytometer (BD). Viral loads were determined using the NucliSENS EasyQ HIV-1 v2.0 kit with a detection limit of 20 copies/ml (BioMérieux, Marcy-l'Étoile, France).

### Quantification of Antiretroviral (ARV) Drugs in Plasma

This study aimed to recruit HIV-1 chronically infected ARV treatment-naïve subjects. ARV therapy usage in chronically infected patients was self-reported. However, certain subjects maintained viral loads below 1,000 RNA copies/ml at the time of assessment. To rule out ARV therapy use, plasma samples were collected from these study participants and analyzed for ARV drugs using a quantitative liquid chromatography coupled with tandem mass spectrometry method. The method screened for nine ARVs commonly available in South Africa at the time of sample collection, namely Emtracitabine, Tenofovir, Lopinavir, Ritonavir, Nevirapine, Abacavir, Lamivudine, Zidovudine, and Efavirenz. A plasma sample volume of 50 μl was processed using a protein precipitation method, ARV drug analytes were chromatographically separated on a Agilent Zorbax Eclipse Plus C18 (2.1 × 50 mm, 3.5 μm) HPLC column (Agilent Technologies, Santa Clara, California, USA), detected using an AB Sciex 5500 triple quadrupole mass spectrometer (Sciex, Framingham, Massachusetts, USA) and quantitated using Analyst® 1.6.2 software (Sciex).

### DARC Genotyping

DARC−46T 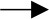 C (rs2814778) single-nucleotide polymorphism (SNP) genotyping was performed by TaqMan allelic discrimination assays which has been previously verified by direct sequence analysis (17). Briefly, genomic DNA was extracted from stored buffy coats using the QIAamp DNA Blood Midi kit (Qiagen, Hilden, Germany) according to manufacturer's instructions. DNA concentration was standardized at 50 ng/μl with polymerase chain reaction (PCR) grade water. A cocktail containing TaqMan Genotyping master mix (Life Technologies, Carlsbad, California, USA) and predesigned probes for the DARC gene (SNP ID: rs2814778, Applied Biosystems, Foster City, California, USA) was used to amplify target sequence in 50 ng genomic DNA by real time PCR in the Light Cycler 480 (Roche, Basel, Switzerland) according to the manufacturer's protocol.

### Neutrophil Isolation

Blood was collected in sodium heparin BD Vacutainers (BD) and processed within 4 h of collection. Granulocyte fraction was prepared by layering equal volumes of whole blood over a Histopaque-1119 cushion (Sigma-Aldrich, St. Louis, Missouri, USA) and centrifuged at 800 × g for 20 min with low brakes as previously described (22). The polymorphonuclear leukocyte band was collected, washed with Dulbecco's phosphate-buffered saline (dPBS) and centrifuged at 300 × g for 10 min, followed by a second wash with chilled wash buffer [dPBS containing 0.5% bovine albumin serum (BSA)] and centrifugation at 300 × g for 10 min at 4°C. Pelleted cells were incubated with magnetically labeled CD15 microbeads (Miltenyi Biotec, Bergisch Gladbach, Germany) for 15 min at 4°C before loading into a MACS column placed in the magnetic field of a MACS separator. Chilled wash buffer was added to the column 3 times. Thereafter, the column was removed from the magnetic field and the retained cells eluted as the CD15+ selected cell fraction, washed once in wash buffer and resuspended at 1 million cells/ml in Roswell Park Memorial Institute (RPMI) 1640 medium without phenol red indicator and containing 0.05% human serum albumin (HSA). Cell counts were determined by 1:5 dilution with Trypan Blue Stain (Gibco) using a hemocytometer under a light microscope. The median neutrophil viability following isolation was 98% (IQR: 98–100%).

### Neutrophil Purity

Purity of the CD15+ cell suspension was assessed by staining with monoclonal antibodies anti-CD66b Fluorescein isothiocyanate (FITC, BD Biosciences) and anti-CD15 Brilliant Violet (BV) 605 (Biolegend, San Diego, California, USA) for 20 min, washed and fixed with Perm Medium A (Invitrogen, Carlsbad, California, USA). Fluorescence minus one (FMOs) for CD66b and CD15 were prepared for each experiment to exclude background fluorescence in the gating strategies. Samples were acquired on an LSRII flow cytometer (BD), recording at least 10,000 events per sample. FlowJo Software Version 9 (TreeStar, Inc., Ashland, Oregon, USA) was used for sample analysis. Only neutrophils with a purity of at least 90% were used for functional assays. The median neutrophil purity was 95.4% (IQR: 91.5–97.8%).

### Phagosome Maturation

The proteolytic activity of neutrophils was evaluated using DQ Green-BSA reporter beads previously developed by Podinovskaia et al. (23). Briefly, preparation of DQ Green-BSA reporter beads was as follows: Carboxylate-modified silica particles (Kisker Biotech GmbH & Co., Steinfurt, Germany) were washed three times with dPBS, resuspended in PBS containing 20 mg cyanamide (Sigma-Aldrich) and incubated on a shaker for 20 min. Washed particles were coupled with DQ Green-BSA (Life Technologies, Carlsbad, California, USA) and 100 μg IgG (Sigma-Aldrich) in coupling buffer (0.1 M boric acid in double distilled water, adjusted to pH 8.0 with 10 M sodium hydroxide) and incubated on a shaker overnight at room temperature. Particles were washed before resuspending in 500 μl dPBS containing 0.02% sodium azide (Sigma-Aldrich) and stored at −20°C in aliquots protected from light (23).

Phagosome maturation was measured in 1 million isolated neutrophils after addition of 5 μl prepared DQ Green-BSA reporter beads and incubation at 37°C for either 10, 60, or 120 min. Samples were fixed with paraformaldehyde (1% final concentration, Sigma-Aldrich) and acquired on an LSRII flow cytometer (BD), recording at least 50,000 events. For each experiment, a tube containing neutrophils incubated for 120 min without DQ Green BSA was acquired for gating strategies and a tube containing DQ Green-BSA reporter beads alone was acquired to determine background fluorescence. Flow cytometry standard files were analyzed using FlowJo Software Version 9 (TreeStar, Inc., Ashland, Oregon, USA).

### ROS Production

ROS production by neutrophils was measured by chemi-luminescence as previously described (24). Briefly, 1 × 10^5^ isolated neutrophils in RPMI-1640 medium containing 50 μM luminol (Sigma-Aldrich) and horseradish peroxidase (Sigma-Aldrich) at a final concentration of 1.2 units/ml were seeded per well in a 96 well plate and allowed to rest at 37°C and 5% CO_2_ for 30 min. Thereafter, cells were cultured with either RPMI-1640 medium alone as a negative control or with 10 nM phorbol 12-myristate 13-acetate (PMA, Sigma-Aldrich) in triplicate and luminescence was read every 2 min for a period of 120 min in a Glomax Multi Detection System (Promega, Madison, Wisconsin, USA). Triplicate readings for unstimulated and PMA stimulated conditions were averaged. Unstimulated readings were used to subtract background luminescence from PMA stimulated readings.

### NET Quantification

A fluorescent microscopy based technique to quantify NET production following neutrophil activation with PMA stimulus was used as previously described (22). Fifty thousand neutrophils in 400 μl RPMI supplemented with 0.05% HSA were seeded on 14 mm coverslips placed in 24 well plates. Neutrophils were either left untreated as a negative control (100 μl RPMI) or activated with 50 nM PMA and incubated at 37°C and 5% CO_2_ for 10, 60, or 120 min before addition of paraformaldehyde fixative solution (2% final concentration) to halt further NET formation. Plates were stored at 4°C overnight until coverslip staining.

Coverslips were washed three times by floating coverslips on dPBS drops placed on parafilm. Cells were permeabilized with 0.5% Triton X-100 (Sigma-Aldrich) in dPBS for 5 min, and blocked with buffer containing 3% donkey serum, 3% fish gelatin, 0.5% Tween (all from Sigma-Aldrich), 1% BSA (Biowest, Nuaillé, France) in dPBS for 30 min. Antibody directed against the subnucleosomal complex of Histone 2A, Histone 2B, and chromatin (mouse anti-PL2-3, Zychlinsky Lab, Max Planck Institute for Infection Biology, Berlin, Germany) and rabbit anti-neutrophil elastase (Calbiochem, San Diego, California, USA) were diluted in blocking buffer, applied to the coverslips and placed in a humid chamber for 1 h in the dark. Coverslips were washed three times with dPBS, and a solution containing Hoechst 33342, dye-conjugated goat anti-mouse Alexa Flour 568 and goat anti-rabbit Alexa Flour 488 (all from Life Technologies) diluted in blocking buffer, was applied to the coverslips and placed in a humid chamber for 45 min in the dark. Coverslips were washed twice with dPBS and once with distilled water before mounting with Mowiol solution (Calbiochem) to glass slides.

NET production was quantified as previously described (25). Images were taken from five randomly selected regions of each coverslip using the 10X lens on a Zeiss upright fluorescence microscope equipped with an AxioCam MRC microscope camera (Oberkochen, Germany). Images were loaded separately for each channel onto ImageJ/FIJI software. The Hoechst 33342 fluorescent channel image was used to collect data of total cell number in a selected region. The image was converted to an 8-bit image and binarised using Bernsen's thresholding method with the parameter 1 contrast threshold value set at 15. Particles were analyzed with the size set to a minimum of 20 pixels. The PL2-3 fluorescent channel image was used to collect data of NET production in the selected region. The threshold was applied to an image fixed with cells that were activated for 10 min with PMA. The threshold was adjusted to the minimum value that allowed only objects larger than 75 pixels (spontaneous NETs > 75 pixels) to be visible. This minimum threshold value was then applied to coverslip regions stimulated for different time intervals for the same specimen. Particle analysis was set with a cut-off-size of 75 pixels. NET production was calculated as follows:

NET-rate (%) = 100 × Objects counted (PL2-3 channel)/Objects counted (Hoechst channel).

The NET production on each coverslip was calculated as an average of the five randomly selected regions. RGB-merged images of the three channels Hoechst, PL2-3 and neutrophil elastase were compiled and used as control images to manually visualize and exclude the possible influence of non-neutrophil cells and artifacts from the results.

### Statistical Analysis

GraphPad Prism Version 5 software (GraphPad software Inc., La Jolla, California, USA) was used for statistical data analysis. Differences between two studied groups was examined by Mann–Whitney *U*-test. Differences between more than two groups was examined by 1 way ANOVA followed by Bonferroni's multiple comparison test. Differences with a *p* < 0.05 were considered statistically significant. For ROS production, area under curve was calculated with the baseline y value = 0 and all values above the baseline were taken into consideration.

## Results

To assess the possible influence of the DARC-null trait and lower ANCs on neutrophil effector functions, 42 participants of Zulu/Xhosa ethnicity were enrolled into our study cohort. The cohort comprised 20 HIV uninfected donors and 22 antiretroviral therapy-naïve HIV-1 chronically infected subjects. The majority of the subjects (40 out of 42) were female with a median age of 20, interquartile range (IQR) (19–22) and 22 years, IQR (20-25) in HIV negative and HIV infected individuals, respectively.

Assessment of the participant cohort by HIV status indicated that CD4 counts were significantly lower in HIV-1 infected persons compared to HIV negative individuals (median CD4 count of 653 vs. 892 cells/mm^3^, respectively, *p* = 0.002, data not shown). Further, analysis by DARC status showed no significant differences in CD4 counts in HIV negative subjects (DARC-null median CD4 count of 824 cells/mm^3^; DARC-positive median CD4 count of 1,198 cells/mm^3^, *p* = 0.08, Table 1), or HIV infected individuals (DARC-null median CD4 count of 582 cells/mm^3^; DARC-positive median CD4 count of 618 cells/mm^3^, *p* = 0.53, Table 1). Furthermore, viral loads were not statistically different by DARC genotype in HIV-infected individuals (DARC-null median viral load of 4,150 RNA copies/ml; DARC-positive median viral load of 12,250 RNA copies/ml, *p* = 0.48, Table 1).

**Table 1:** Clinical characteristics of study participants.

	**HIV negative**			**HIV Positive**		
	**DARC CC (*n* = 13)**	**DARC TC/TT (*n* = 7)**	***p*-value**	**DARC CC (*n* = 16)**	**DARC TC/TT (*n* = 6)**	***p*-value**
Absolute neutrophil count, 10^3^ cells/mm^3^	**2.64** (1.95–3.17)	**5.07** (4.68–7.08)	0.0007	**2.22** (1.78-2.66)	**4.08** (3.13-4.78)	0.03
CD4 count, cells/mm^3^	**824** (624–1,170)	**1,198** (702–1,471)	0.08	**582** (457–693)	**618** (471–799)	0.53
Viral load, RNA copies/ml	na	na	na	**4,150** (1,175–30,750)	**12,250** (3,750–24,500)	0.48

*Data is represented as median values in bold and IQR in brackets. DARC, Duffy Antigen Receptor for Chemokines; DARC CC, DARC-null; DARC TC/TT, DARC-positive; n, number; na, not applicable; IQR, Interquartile range. Female participants: n = 40, Male participants: n = 2*.

### High Prevalence of the DARC-Null Trait in the Zulu/Xhosa Population

The DARC-null trait is exceedingly prevalent on the African continent (10). Prevalence of this polymorphism has previously been reported in persons of Zulu/Xhosa ethnicity at 64.8% in HIV-infected individuals and at 64.7% in high risk South African women (13, 17). Here we used allelic discrimination assays for SNP genotyping in order to distinguish DARC-null and DARC-positive individuals. Twenty nine of the 42 participants (69%) were negative for the DARC trait irrespective of HIV status. In HIV negative individuals, 13 of 20 participants (65%) were DARC-null, whilst 7 of 20 (35%) were DARC-positive. In HIV-infected subjects, the presence of the polymorphism was even higher, 16 of 22 participants (73%) were DARC-null and 6 of 22 subjects (27%) were DARC-positive (Table 1). Thus, despite the comparatively smaller sample size used here, our data is in agreement with past studies (13, 17) that indicate high prevalence of the DARC-null allele in populations within this region.

### DARC-Null Trait Is Associated With Reduced ANCs

Neutrophils constitute 50–70% of circulating leukocytes with typical counts ranging from 2.0 × 10^3^ to 7.5 × 10^3^ cells/mm^3^ of whole blood. Previous reports have indicated a strong association of the DARC variant with ANCs. The DARC-null polymorphism is accompanied with persistently lower concentrations of circulating neutrophils and is a predictor of ethnic neutropenia where ANCs are repeatedly <1.5 × 10 cells/mm^3^ (7). To ascertain whether our study cohort displayed similar associations, we compared ANCs according to DARC trait in both uninfected and HIV-1 infected individuals.

**Figure 1:**
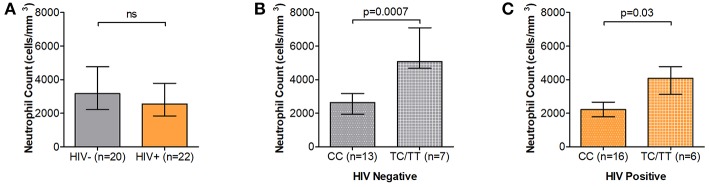
Absolute neutrophil Counts (ANCs) stratified by DARC variant. **(A)** Comparison of ANCs measured by full blood count in HIV negative and HIV positive individuals. ANCs were further stratified according to DARC genotype in HIV negative **(B)** and HIV positive **(C)** individuals. Bar graphs indicate medians extended to interquartile range (IQR) with whiskers. The *p*-values refer to differences in ANC between DARC-null and DARC-positive participants at the time of sampling. ANC, Absolute Neutrophil Count; DARC, Duffy Antigen Receptor for Chemokines; CC, DARC-null; TC/TT, DARC-positive; IQR, interquartile range; ns, *p*-value not significant.

Among our study participants we found no differences in ANCs by HIV status where median ANCs were 3.17 × 10^3^ and 2.56 × 10^3^ cells/mm^3^ in HIV uninfected and HIV-infected individuals, respectively (Figure 1A). We did, however, observe significantly reduced ANCs in DARC-null compared to DARC-positive individuals, irrespective of HIV status, although this finding was more prominent in uninfected individuals. In uninfected individuals, a median ANC of 2.64 × 10^3^ cells/mm^3^ was observed in DARC-null subjects compared to 5.07 × 10^3^ cell/mm^3^ in DARC-positive subjects (*p* = 0.0007, Table 1, Figure 1B). In HIV-1 chronically infected individuals, a median ANC of 2.22 × 10^3^ cells/mm^3^ was observed in DARC-null subjects compared to 4.08 × 10^3^ cells/mm^3^ in DARC-positive subjects (*p* = 0.03, Table 1, Figure 1C). Despite the high prevalence of the Duffy-null trait in our cohort, only 4 of 29 of these individuals (13.8%) had neutropenia, defined as ANCs of 1.5 × 10^3^ cells/mm^3^ or less at the time of sampling (data not shown). Overall, in line with current literature (11), DARC-null individuals exhibited significantly lower circulating neutrophil counts, albeit the proportion of individuals meeting the definition of ethnic neutropenia was low.

### Neutrophil Proteolytic Activity Enhanced in Individuals With DARC-Null Trait

**Figure 2:**
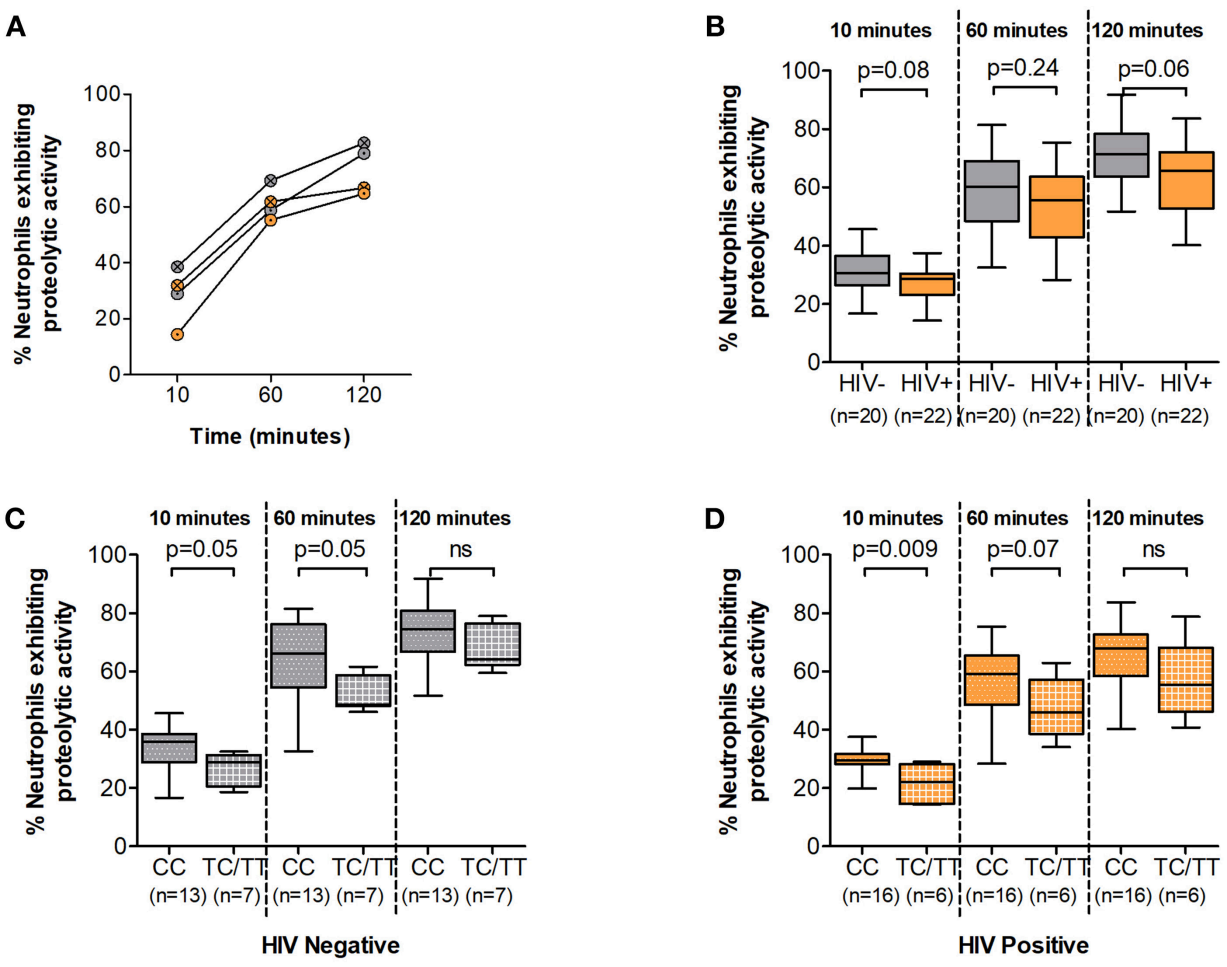
Neutrophil phagosome maturation. **(A)** Representative examples of Neutrophil Phagosome Maturation time course. HIV negative individuals are represented as gray circles; HIV positive individuals are represented as orange circles. Circles are further indicated with symbols (X) for DARC-null or (·) for DARC-positive individuals, respectively. **(B)** Comparison of phagosome proteolytic activity by HIV status. Proteolytic activity was further assessed by DARC status in HIV negative **(C)** and HIV-infected **(D)** individuals, respectively. The *p*-values indicate differences in proteolytic activity between subject groups. Boxes extend from the 25th to 75th percentile and medians are indicated by the horizontal line. Whiskers indicate minimum and maximum values. DARC, Duffy Antigen Receptor for Chemokines; CC, DARC-null; TC/TT, DARC-positive; ns, *p*-value not significant.

Recognition, internalization and phagosome maturation are the hallmarks of the neutrophil response to effectively clear invading pathogens (26). We thus sought to measure the phagosome maturation of neutrophils following Fc mediated phagocytosis in DARC-null individuals and whether this proteolytic activity is altered by DARC status or ANCs in HIV-1 uninfected and HIV-1 infected individuals. Here we measured phagosome maturation by flow cytometry after 10, 60, and 120 min of exposing IgG opsonized beads to isolated neutrophils (Supplementary Figure 1). Phagosome maturation exhibited a steady increase over all time intervals in the 120 min period in all studied subjects, and this increase was irrespective of HIV-1 status (Figure 2A and Supplementary Figure 2, *p* < 0.0001). HIV-1 infection has been reported to reduce neutrophil phagocytosis capacity, although the mechanisms underlying this altered activity are not fully understood (6). Here a trend of lower neutrophil proteolytic activity in HIV chronically infected compared to uninfected persons was observed at all measured time intervals over a 120 min period (Figure 2B, *p* = 0.08 at 10 min; *p* = 0.24 at 60 min; *p* = 0.06 at 120 min).

Subsequent analysis by DARC status revealed that DARC-null individuals (irrespective of HIV status) had a higher proportion of neutrophils exhibiting phagosome maturation at 10 and 60 min (Figure 2C, *p* = 0.05 at 10 min, and *p* = 0.05 at 60 min in HIV negative individuals; and Figure 2D, *p* = 0.009 at 10 min, and *p* = 0.07 at 60 min in HIV infected individuals). The median frequency of neutrophils exhibiting proteolytic activity was still higher in DARC-null compared to DARC-positive individuals at 120 min post bead incubation (74.5 vs. 64.3% in HIV uninfected and 67.9 vs. 55.4% in HIV infection in DARC-null and DARC-positive individuals, respectively). However, overall no statistical differences were observed in the proteolytic activity potential between DARC-null and DARC-positive individuals in HIV uninfected or HIV infected persons at 120 min post bead incubation (Figures 2C,D). In HIV uninfected individuals, the frequency of neutrophils exhibiting proteolytic activity did not correlate with ANCs; in contrast, the number of neutrophils displaying proteolytic activity inversely correlated with ANCs in chronically infected individuals at 10 min (a trend was observed, *p* = 0.06, data not shown) and 60 min (*p* = 0.04, data not shown) following bead incubation.

Taken together our data suggests that the DARC genotype may affect the proteolytic activity potential of neutrophils irrespective of HIV status. In the absence of the DARC phenotype, a higher proportion of neutrophils exhibited phagosome maturation soon after bead incubation (10 and 60 min).

### ROS Production Is Unaffected by DARC Status

**Figure 3:**
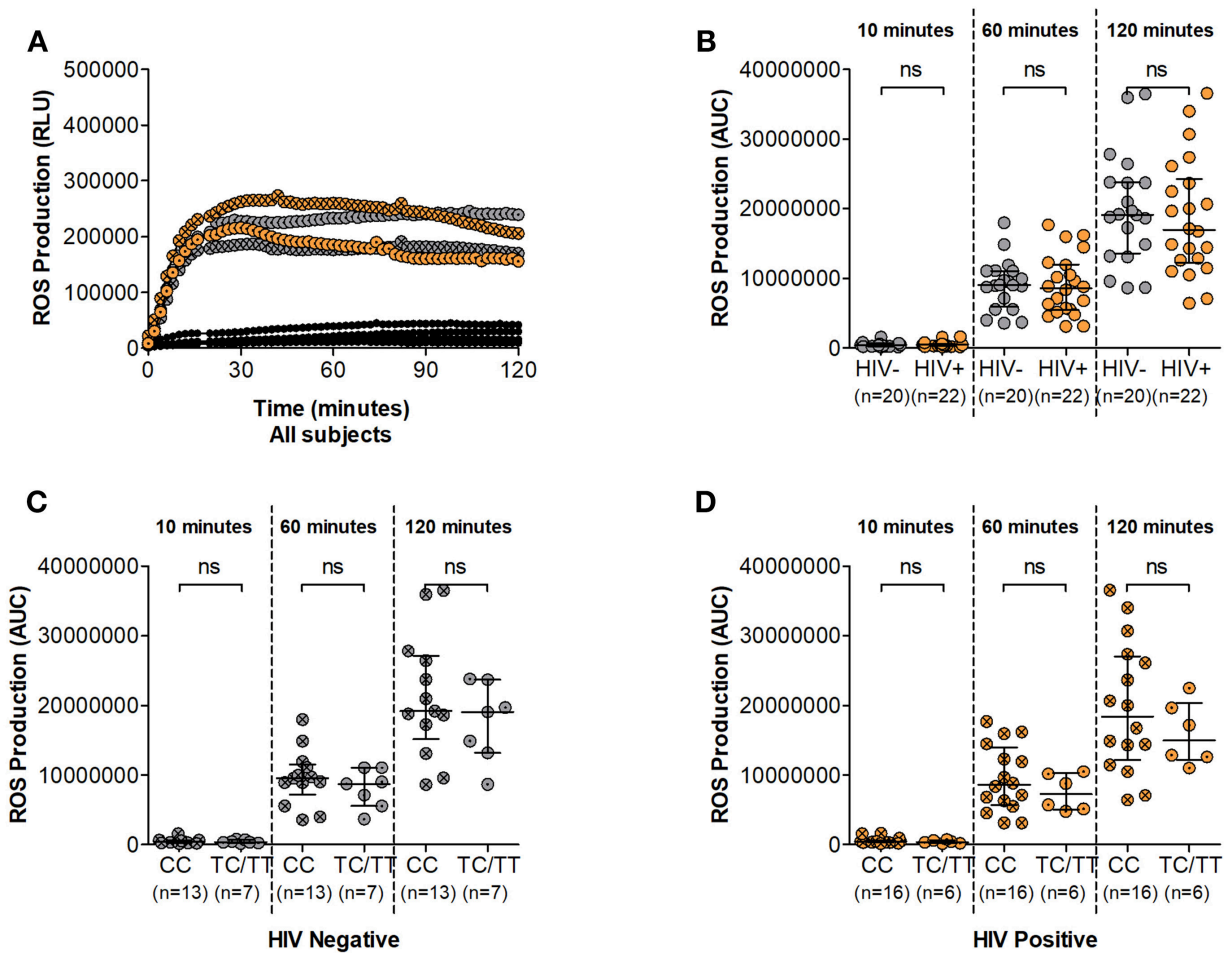
Neutrophil Reactive Oxygen Species (ROS) production. **(A)** Representative examples of ROS production time course. Unstimulated controls are represented as black circles; HIV negative individuals are represented as gray circles; HIV positive individuals are represented as orange circles. Circles are further indicated with symbols (X) for DARC-null or (·) for DARC-positive individuals, respectively. ROS production is measured as Relative Light Units (RLU). **(B)** Comparison of Neutrophil ROS production by HIV status. Neutrophil ROS production was further assessed by DARC status in HIV negative **(C)** and infected **(D)** individuals, respectively. The quantitative measure of ROS production is presented as area under the curve (AUC). Dots indicate individual data points. Medians are indicated and extended to interquartile range (IQR) with whiskers. The *p*-values as indicated by ns refer to no significant differences by HIV or DARC status. ROS, reactive oxygen species; RLU, Relative Light Units; AUC, Area Under Curve; DARC, Duffy Antigen Receptor for Chemokines; CC, DARC-null; TC/TT, DARC-positive; IQR, interquartile range; ns, *p*-value not significant.

During the phagocytic process several enzymatic mechanisms are involved in microbial killing. Superoxide burst leads to the release of toxic compounds within the phagosome and is crucial to these processes. We next assessed the superoxide producing potential of neutrophils from study participants following *in vitro* PMA stimulation. Luminol oxidation measured by chemi-luminescence was used as an indicator of ROS production. Similar to other effector mechanisms, the ability of neutrophils to generate ROS has been shown to be compromised during HIV infection (6). Here, ROS release was observed within 10 min of PMA stimulation and reached peak secretion within 30 min irrespective of HIV status (Figure 3A and Supplementary Figure 3). ROS production was higher in HIV negative compared to infected persons at 60 and 120 min (median luminol oxidation was measured at 9.04 × 10^6^ at 60 min and 1.91 × 10^7^ Area Under the Curve (AUC) at 120 min compared to 8.59 × 10^6^ at 60 min and 1.70 × 10^7^ AUC at 120 min in HIV negative and infected individuals, respectively). However, statistical analysis showed no significant differences between HIV uninfected and infected individuals at 10, 60, or 120 min following stimulation (Figure 3B).

Further assessment indicated higher median ROS production in DARC-null compared to DARC-positive participants regardless of HIV status. In HIV uninfected individuals, median luminol oxidation was measured at 9.53 × 10^6^ and 1.92 × 10^7^ AUC in DARC-null compared to 8.74 × 10^6^ and 1.91 × 10^7^ AUC in DARC-positive persons at 60 and 120 min, respectively. In infected individuals, median luminol oxidation was measured at 8.62 × 10^6^ and 1.84 × 10^7^ AUC in DARC-null compared to 7.26 × 10^6^ and 1.50 × 10^7^ AUC in DARC-positive persons at 60 and 120 min, respectively. Nevertheless, statistical analysis displayed no significant disparities in overall superoxide release according to DARC status (Figures 3C,D) or by ANCs (data not shown) in uninfected or infected persons.

Taken together our data suggests that the potential of neutrophils to produce ROS is unaffected by HIV infection in this study population. This was unexpected as it was in contrast to previous reports of diminished ROS production in HIV infected individuals (6). Furthermore, our results indicate that neither the absence of DARC nor lower ANCs (when neutrophil numbers are standardized) impact neutrophil ROS release.

### Prolonged Exposure to Stimulus Leads to Lower NET Formation in DARC-Null Persons During HIV Infection

**Figure 4:**
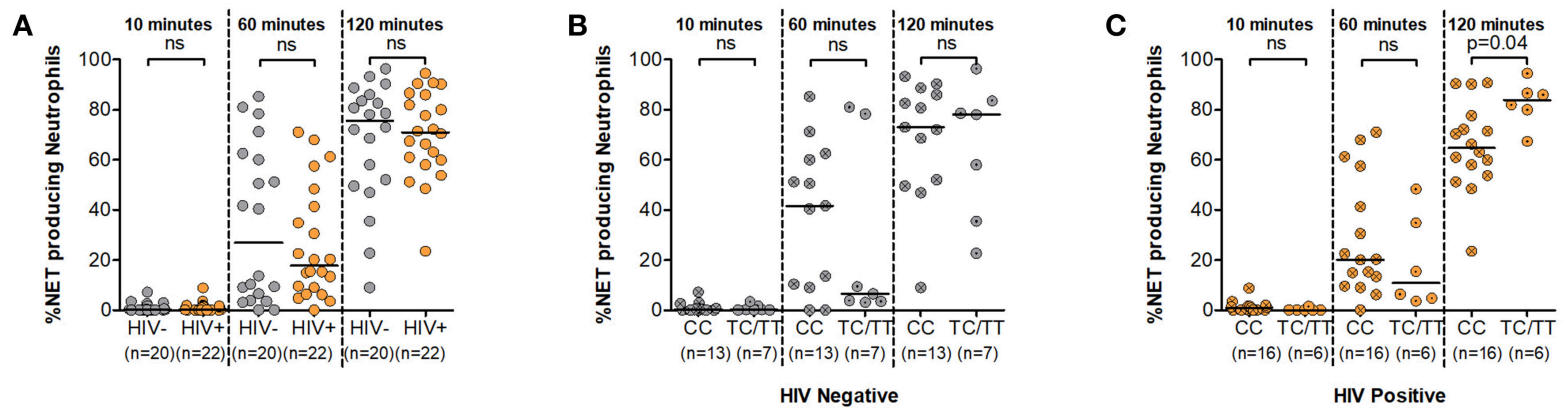
Neutrophil Extracellular Trap (NET) production. **(A)** Comparison of NET production by HIV status. NET production was further assessed by DARC status in HIV negative **(B)** and HIV-infected **(C)** individuals, respectively. Dots indicate individual data points. Medians are indicated as horizontal lines. The *p*-values refer to differences in NET production between subject groups. NET, Neutrophil extracellular trap; DARC, Duffy Antigen Receptor for Chemokines; CC, DARC-null; TC/TT, DARC-positive; ns, *p*-value not significant.

The ability of neutrophils to form NETs in a process called NETosis is a relatively recent discovery (27). It involves the release of genomic material together with antimicrobial factors in an effort to counter larger pathogens that evade phagocytosis (28). Here we measured NETosis following neutrophil stimulation with PMA over a 120 min period using fluorescent microscopy (Supplementary Figure 4). Data demonstrating NET quantification during HIV infection is lacking and also it is unknown whether DARC genotype impacts NET production. To address this gap, we tested the hypothesis that NET production upon neutrophil activation is altered in the absence of DARC and that this impairment would be markedly pronounced during HIV infection. Our findings demonstrated higher median frequencies of NET producing neutrophils in HIV uninfected persons compared to HIV infected individuals over time, however no significant differences were observed by HIV status in our study (Figure 4A). The median frequency of NET producing neutrophils was 27% at 60 min and 76% at 120 min in HIV negative individuals compared to 18% at 60 min and 71% at 120 min in HIV infected persons (Figure 4A).

Assessment of this function by DARC status indicated greater median frequencies of NET production in the absence of DARC at the 10 and 60 min time intervals in both uninfected and infected subjects. The median frequency of NET producing neutrophils was 0.3 and 42% in DARC-null compared to 0.2 and 7% in DARC-positive persons at 10 and 60 min post stimulation in HIV uninfected individuals. In chronically infected persons, the median frequency of NET producing neutrophils was 1 and 20% in DARC-null compared to 0.03 and 11% in DARC-positive subjects at 10 and 60 min post stimulation, respectively. However, statistical analysis did not show any significant differences in NET production between DARC-null and DARC-positive individuals at 10 or 60 min post stimulation irrespective of HIV status (Figures 4B,C). In contrast, following prolonged exposure to stimulus (120 min), DARC-null individuals had lower NET production medians compared to DARC-positive persons. Median frequencies of NET producing neutrophils was 73% in DARC-null compared to 78% in DARC-positive HIV uninfected subjects. During HIV infection, median NET production was 65% in DARC-null compared to 84% in DARC-positive individuals (Figures 4B,C). Moreover, overall examination by DARC status revealed significantly lower NET release in DARC-null chronically infected persons compared to their DARC-positive counterparts (Figure 4C, *p* = 0.04). Further analyses revealed no association between the frequency of NET producing neutrophils and ANCs regardless of HIV or DARC status (data not shown).

Taken together this data suggests that neutrophils are not significantly impaired in their ability to produce NETs during HIV infection. Furthermore, neutrophils from DARC-null individuals are rapidly activated within the first hour to produce NETs in response to stimulus. However, during chronic HIV infection, prolonged activation of neutrophils may lead to impairment of NETosis activity in DARC-null individuals.

## Discussion

DARC-null linked neutropenia is associated with increased susceptibility to HIV acquisition. Interestingly, subsequent to infection, reports suggest a survival advantage imparted on leukopenic DARC-null persons in comparison to non-leukopenic DARC-null and DARC-positive individuals. However, the mechanisms involved in these associations are not fully elucidated. We investigated the influence of DARC on neutrophil functionality in HIV negative and therapy-naïve HIV infected individuals in a DARC-null prevalent population from Durban, South Africa.

The DARC-null polymorphism was highly prevalent in our cohort and we observed that ANCs were reduced in individuals with the DARC-null trait regardless of HIV status. The DARC-null trait is predominantly displayed in persons of African ancestry, and the absence of DARC has been associated with lower neutrophil counts in circulation (11, 12). Assessment of HIV-1 chronically infected African individuals based in Durban indicated that 64.8% (247 of 381) of the patients were homozygous for the DARC-null allele (17), whilst a longitudinal study of high risk South African woman described that 64.7% (92 of 142) of their study subjects possessed the DARC-null trait (13). We here observed comparable frequencies of the DARC-null variant (69%) within a similar population group of Zulu/Xhosa individuals from the Durban area. Furthermore, in accordance with previous reports, the DARC-null genotype was significantly associated with reduced neutrophil counts in studied individuals irrespective of HIV status, although this finding was substantially less prominent in chronic HIV infection. Accelerated neutrophil apoptosis is generally observed in HIV infection (6). Whether the effect of the DARC-null variant on neutrophil count is overshadowed by this accelerated apoptosis is unclear and requires further investigation.

The functional capacity of neutrophils as assessed by three key effector functions, i.e., proteolytic activity and by producing ROS and NETs demonstrated moderately differential results. A trend of lower proteolytic activity was observed in HIV infected individuals compared to HIV negative subjects; however, neither production of ROS or NETs were altered in HIV infection. Assessment of the neutrophil functions by DARC status, revealed higher proteolytic activity in DARC-null compared to DARC-positive individuals, which was more pronounced in HIV infected than in uninfected subjects. In contrast, a reduction in neutrophil NET production was noted in HIV infected individuals with the DARC-null trait following 2 h of *in vitro* stimulation.

There are conflicting reports of neutrophil responses in HIV-1 disease. While few studies have described an increase of key killing responses in HIV-1 infected patients compared to their uninfected counterparts (29, 30), the general consensus suggests that infection leads to defective neutrophil responses and a higher rate of neutrophil apoptosis (6). Past studies have reported significantly impaired neutrophil phagocytosis and reduced superoxide burst in HIV-1 infected individuals (6). Furthermore, superior neutrophil functional responses were detected in patients with better immunological status and lower viral loads (31). While the mechanism for altered phagocytic activity remains unclear, reports of monocyte function suggest that HIV proteins impede phagocytosis through downregulation of the gamma signaling chain of the Fc receptor (32) and inhibit phagosome formation in a Nef-dependent manner (33). In agreement with earlier reports, we observed lower phagosome maturation following FcR-mediated phagocytosis in HIV infected persons compared to HIV negative subjects, although the difference was not significant but rather a strong trend (*p* = 0.08 at 10 min and *p* = 0.06 at 120 min).

We also found that HIV-infected individuals did not display lower ROS production in comparison to uninfected individuals. Plasma RNA levels have been shown to be associated with reduced ROS production. Individuals with <1,000 viral copies/mL have been shown to display unaltered oxidative metabolism. Reduced ROS release was detected in patients with more than 1,000 viral copies/ml, while extreme ROS dysfunction was observed when viral load exceeded 10,000 viral copies/ml (34), signifying the direct role of HIV virions in the alteration of ROS release (35). Moreover, no significant modification of ROS production was described in HIV-1 infected asymptomatic individuals with a CD4 count above 200 cells/mm^3^ (35).

Importantly, these previous reports noted that the severity of neutrophil impairment is dependent on the stage of HIV disease. Neutrophil dysfunction is more evident in the later stages of infection in patients with high HIV-1 plasma concentrations and lower CD4 lymphocyte counts (36, 37). Moreover, the prevalence of coinfection with opportunistic pathogens during advanced disease contributes to diminished neutrophil response (38). Chronically infected subjects that were studied here presented with low viral loads at the time of sample collection, 59% (13 of 22) had a viral load lower than 10,000 copies/ml and all HIV infected persons studied had a CD4 count above 300 cells/mm^3^. Clinically, these data indicate that none of these individuals were in advanced stages of disease, and could partly explain our observation of no significant differences in proteolytic capacity or levels of ROS production between HIV uninfected and infected individuals.

Furthermore, our data suggests that NET release following PMA exposure was unaffected in HIV infection; it is plausible that variations in NET production due to HIV may not have been detected due to lower viral loads in our studied cohort. In contrast to other neutrophil functions, data on NET formation is lacking with regard to HIV infection. The role mediated by NET formation in preventing HIV transmission has been described (39, 40), however, whether this mechanism is modified following infection remains unclear.

Various genetic mutations have been associated with neutrophil disorders either in count and/or function (41, 42). It is well-established that the absence of the DARC phenotype is associated with lower circulating ANCs characteristic of ethnic neutropenia. Yet the influence of this polymorphism on neutrophil function has been virtually unexplored. A recent study reported similar neutrophil gene expression in subjects with and without ethnic neutropenia, suggesting intact neutrophil function in individuals with low DARC expression (43).

We assessed the impact of the DARC-null polymorphism on neutrophil effector functions; and noted enhanced phagosome maturation in neutrophils from DARC-null individuals in the first hour and this activity was not adversely affected by HIV infection. Neutrophil phagosome maturation following FcR engaged phagocytosis is characteristically very rapid, and efficient phagosome formation is a clear advantage in terms of host immunity (26, 44). A recent report indicated that DARC knockout murine models exhibited phenotypically distinct neutrophils, displaying higher expression of FcγR and CD45, which is known to enhance FcγR function (45). Similarly, neutrophils characterized from uninfected DARC-negative individuals were found to selectively upregulate FcγRIIIb (CD16) and CD45 expression (45). We show higher proteolytic activity potential in neutrophils from DARC-null individuals, specifically at the earlier time intervals. Whether this enchanced activity arose from higher FcR-mediated phagocytosis or specific factors within the phagosome is uncertain and warrants further investigation. It is possible that this elevated proteolytic activity is a consequence of augmented FcR expression which resulted in higher phagocytosis of IgG opsonized beads. Nevertheless, this data suggests faster clearance of pathogens and thus better outcomes in DARC-null individuals.

Furthermore, we observed higher (although not significant) frequencies of neutrophils able to produce ROS and NETs in DARC-null individuals within the first hour following stimulation regardless of HIV status. Neutrophil activation progresses through multiple steps, and neutrophils are considered to be fully activated when they are able to fully function. Responsiveness to stimuli priming allows for rapid and efficient neutrophil activation (26). The data may suggest that neutrophil activation is more easily induced in DARC-null persons, resulting in a higher frequency of cells with earlier responses to stimulus compared to DARC-positive individuals. Interestingly, gene expression studies indicated marginally activated neutrophil profiles in DARC-null individuals with benign ethnic neutropenia (43). Whilst leukocyte migration and hematopoietic stem cell mobilization pathways were most affected (43), this slightly activated state of neutrophils from DARC-null persons could contribute to rapid induction of neutrophil defense mechanisms.

While earlier functional responses (within the first hour) to stimulus were seemingly slower in neutrophils from DARC-positive individuals, prolonged stimulation (2 h) resulted in neutrophil activity that was comparable to neutrophils from DARC-null neutrophils. An exception to this observation was a more robust NET response following prolonged stimulation in neutrophils from DARC-positive HIV infected individuals compared to their DARC-negative counterparts. Interestingly, this variation in NET release was not observed in HIV uninfected individuals according to DARC genotype, suggesting that chronic inflammation during HIV infection may contribute to lower NET production in DARC-null HIV infected persons. Slightly activated neutrophil profiles exhibited in the absence of DARC coupled with continued inflammation during HIV infection could induce faster neutrophil cell exhaustion and death in DARC-null persons. While diminished NET production would be unfavorable in clearing pathogens, it has been noted that disproportionate NET formation occurring in chronic activation elicits antibody-mediated autoimmune activity and organ damage (46, 47). Thus, lower NET formation in DARC-null chronically infected persons may be beneficial in averting excessive tissue damage in these individuals.

While the data presented here provides evidence of moderate variation in neutrophil function in the absence of the DARC allele, we were limited by numerous factors. Neutrophils are fragile and the 4 h period between blood collection and processing was longer than the ideal conditions when assessing neutrophil activity; however, this time period was consistent for all samples. We were also limited by the sample volume per participant and were thus restricted by the number of isolated neutrophils and the functional assays that could be conducted. Diverse approaches to compliment the functional data presented here would benefit future studies. These complimentary assays could include analysis of neutrophil functional kinetics over a longer time period and in response to various physiological agonists. Other limitations included a small sample size and chronically infected patients with relatively low viral loads. Our observations may therefore not be representative of typical disease progression in the absence of antiretroviral therapy. Furthermore, many of our DARC-null subjects were not neutropenic as defined by the ANC threshold of <1,500 cells/mm^3^, and since these patients were not followed longitudinally we cannot determine which patients possessed neutropenic episodes. Additional sample collection from chronically infected treatment naïve individuals became challenging during the study due to the global implementation of the HIV test and treat policy.

Despite these limitations, we were able to identify variation in neutrophil responses. We noted differences in neutrophil phagosome maturation as either an effect of HIV infection or lack of DARC. Although participants with identified ethnic neutropenia were scarce, we were able to provide evidence of the influence of the DARC-null polymorphism rather than lower ANCs on neutrophil effector functions irrespective of HIV status. Whilst our study demonstrates no impairment of neutrophil function at the single cell level, it is important to note that the elimination of pathogens requires cooperativity of multiple effector cells, and it is possible that neutropenic individuals would still have reduced capability to clear infection *in vivo* due to insufficient number of neutrophils.

In summary, we addressed the impact of the DARC-null allele on three key neutrophil responses in persons recruited from an HIV-1 prevalent population. We focused on these three functions as they are primary to the killing efficiency of neutrophils as a first line of host immunity. Beyond these effector functions, neutrophils have emerged as immunomodulatory cells that contribute to ongoing immune responses through either soluble protein secretion or direct interaction with other immune cells (26), and these mechanisms in the context of DARC require future attention.

To our knowledge this is the first study to assess the influence of the DARC-null polymorphism on neutrophil function in an HIV-1 setting. Our findings are mostly contrary to our hypothesis of impaired neutrophil responses in the absence of DARC. Neutrophils from DARC-null individuals with lower ANCs were not functionally impaired. Interestingly, our data suggested that neutrophils displayed higher proteolytic activity in the absence of DARC which could suggest a compensatory mechanism for lower ANCs in these individuals. Alternatively, a possible switch between proteolytic activity and NETosis mechanisms cannot be ruled out and warrant further investigation. Superior proteolytic activity associated with the DARC-null trait could explain the benign, asymptomatic characteristics associated with ethnic neutropenia. Furthermore, higher neutrophil proteolytic activity detected in DARC-null individuals may serve as an advantage in chronic HIV infection, where a higher proportion of neutrophils able to rapidly phagocytose could benefit DARC-null individuals in immune defense against opportunistic infection in stages of advanced disease. Overall, our data provide evidence that the DARC-null allele is not overtly deleterious in relation to neutrophil function in people of African descent.

## Data Availability

All datasets generated for this study are included in the manuscript and/or the Supplementary Files.

## Author Contributions

KN: acquisition of data, execution, interpretation of experiments and results, analysis of the data, and drafting the manuscript; AN, PG, and AM: sample collection and acquisition of data; TN: supervisory support, interpretation of data, and revising the manuscript; CT: conception and study design, supervisory support, interpretation of data, and revising the manuscript.

### Conflict of Interest Statement

The authors declare that the research was conducted in the absence of any commercial or financial relationships that could be construed as a potential conflict of interest.

## Supplementary Material

Supplementary Figure 1 Representative gating strategy for Phagosome Maturation measurement by flow cytometry. Background fluorescence was determined by acquisition of DQ Green-bovine serum albumin (BSA) reporter beads alone. The gating strategy for neutrophils was determined by acquisition of neutrophils without DQ Green-BSA reporter beads (cells only). The gating strategy was applied to neutrophil samples incubated with DQ Green-BSA reporter beads for 10, 60, and 120 min. Proteolytic activity within the phagosome was measured as DQ Green-BSA (Alexa Flour 488-A) positive. BSA, bovine serum albumin.

Supplementary Figure 2 Neutrophil Phagosome Maturation time course. **(A)** Proteolytic activity within the phagosome over time is shown for all individuals where HIV negative individuals are represented as gray circles and HIV positive individuals are represented as orange circles, respectively. **(B)** HIV negative subjects are further represented by DARC status where DARC-null individuals are indicated by gray circles and DARC-positive individuals are represented by white circles. **(C)** HIV positive subjects are further represented by DARC status where DARC-null individuals are indicated by orange circles and DARC-positive individuals are indicated by white circles. Dots indicate data points for each participant joined by lines over the 120 min time interval. The *p*-values refer to the differences in proteolytic activity between all timed intervals. DARC, Duffy Antigen Receptor for Chemokines.

Supplementary Figure 3 Neutrophil Reactive Oxygen Species (ROS) production time course. **(A)** Neutrophil ROS production in all individuals as measured by chemi-luminescence (relative light units, RLU) every 2 min over a 120 min time interval. Black circles indicate unstimulated samples. Stimulated samples are indicated by gray (HIV negative) and orange (HIV positive) circles. **(B)** HIV negative individuals are further presented by DARC status where DARC-null individuals are represented as gray circles and DARC-positive individuals are represented as white circles. **(C)** HIV positive individuals are further represented by DARC status where DARC-null individuals are represented as orange circles and DARC-positive individuals are represented as white circles. Circles indicate data points for each participant joined by lines over the 120 min time interval. ROS, reactive oxygen species; RLU, Relative Light Units; DARC, Duffy Antigen Receptor for Chemokines.

Supplementary Figure 4 Neutrophil Extracellular Trap Microscopy. Representative microscopy images showing Hoechst (blue), anti-PL2-3 (red), and anti-neutrophil elastase (green) staining staining at 10x (top panel) and 20x (bottom panel) magnification after 60 min post PMA activation. Overlaying of the three channels created the merged image. Red boxes indicate the region shown in the 20x magnification panel.
