# Supplementary figures and images for: Neutrophil Effector Functions Are Not Impaired in Duffy Antigen Receptor for Chemokines (DARC)-Null Black South Africans
Source: Front Immunol. 2019 Mar 26;10:551. doi: 10.3389/fimmu.2019.00551 (PMC6443851; doi:10.3389/fimmu.2019.00551)

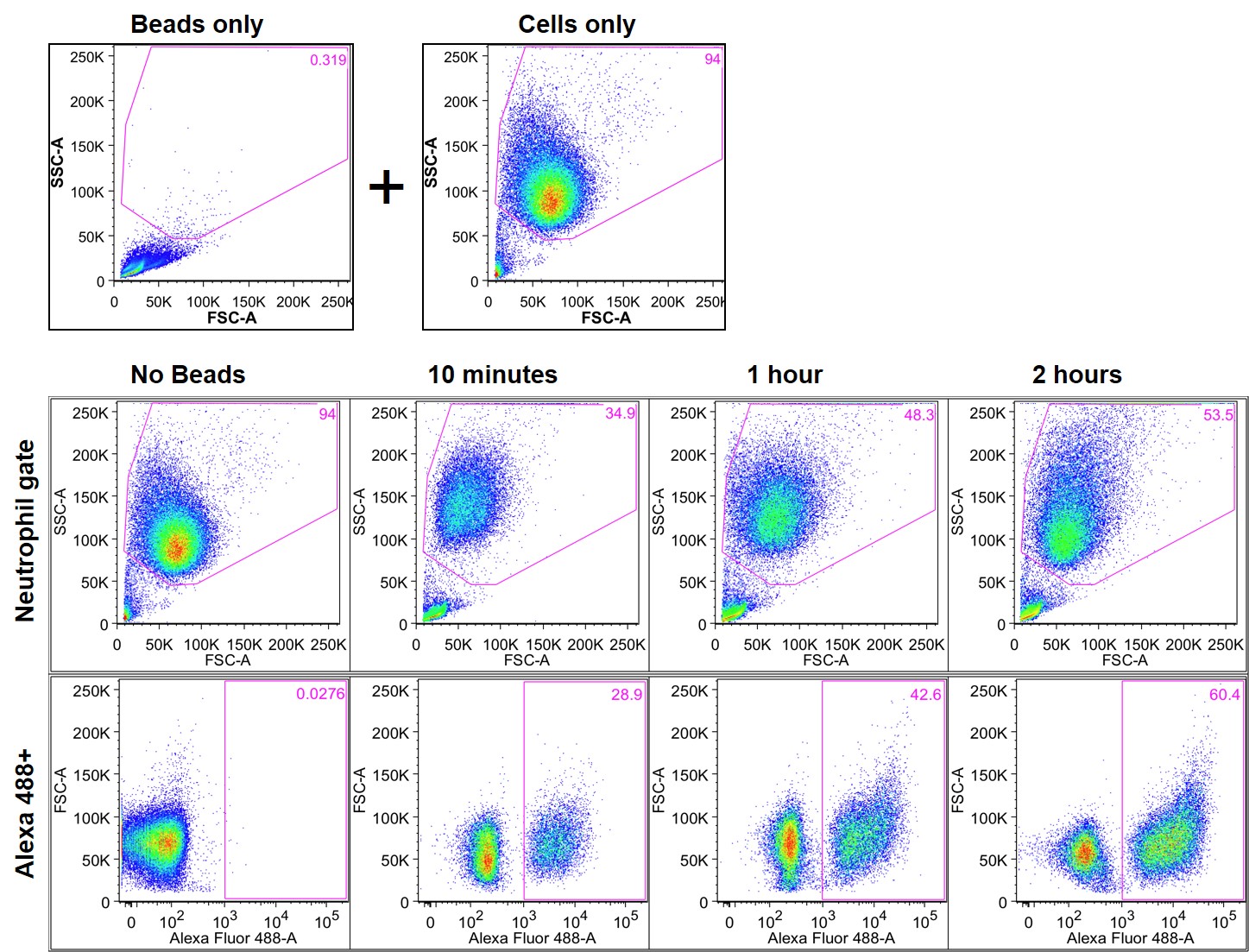

Supplement: Supplementary Figure 1 — Representative gating strategy for Phagosome Maturation measurement by flow cytometry. Background fluorescence was determined by acquisition of DQ Green-bovine serum albumin (BSA) reporter beads alone. The gating strategy for neutrophils was determined by acquisition of neutrophils without DQ Green-BSA reporter beads (cells only). The gating strategy was applied to neutrophil samples incubated with DQ Green-BSA reporter beads for 10, 60, and 120 min. Proteolytic activity within the phagosome was measured as DQ Green-BSA (Alexa Flour 488-A) positive. BSA, bovine serum albumin. [file Image_1.JPEG]

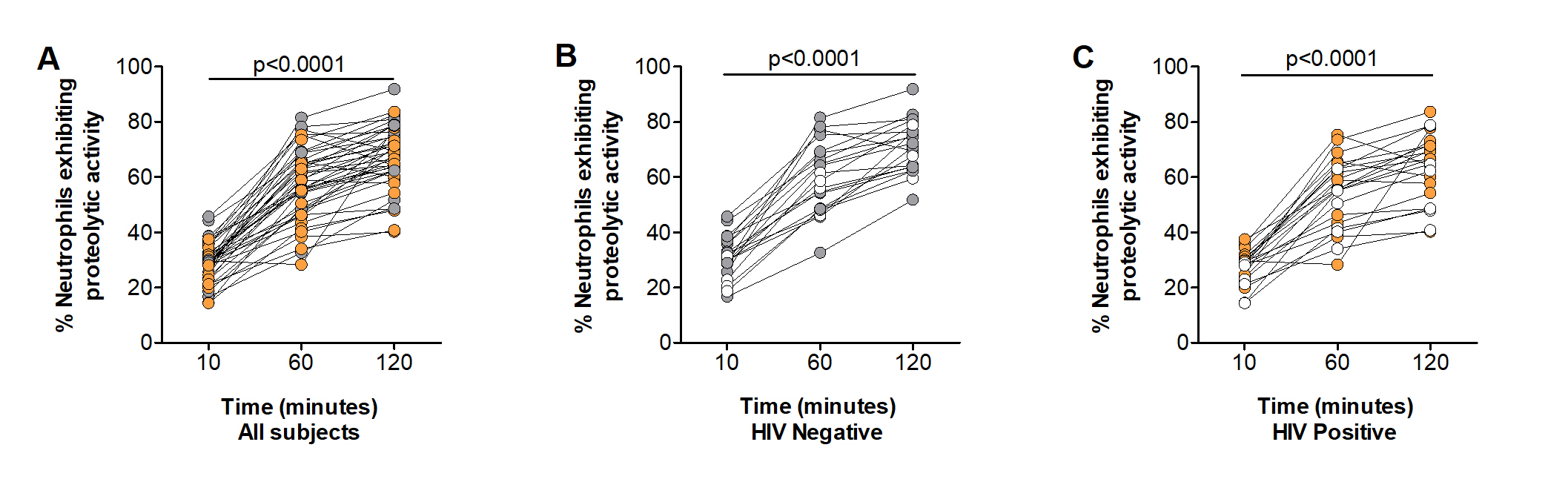

Supplement: Supplementary Figure 2 — Neutrophil Phagosome Maturation time course. (A) Proteolytic activity within the phagosome over time is shown for all individuals where HIV negative individuals are represented as gray circles and HIV positive individuals are represented as orange circles, respectively. (B) HIV negative subjects are further represented by DARC status where DARC-null individuals are indicated by gray circles and DARC-positive individuals are represented by white circles. (C) HIV positive subjects are further represented by DARC status where DARC-null individuals are indicated by orange circles and DARC-positive individuals are indicated by white circles. Dots indicate data points for each participant joined by lines over the 120 min time interval. The p-values refer to the differences in proteolytic activity between all timed intervals. DARC, Duffy Antigen Receptor for Chemokines. [file Image_2.JPEG]

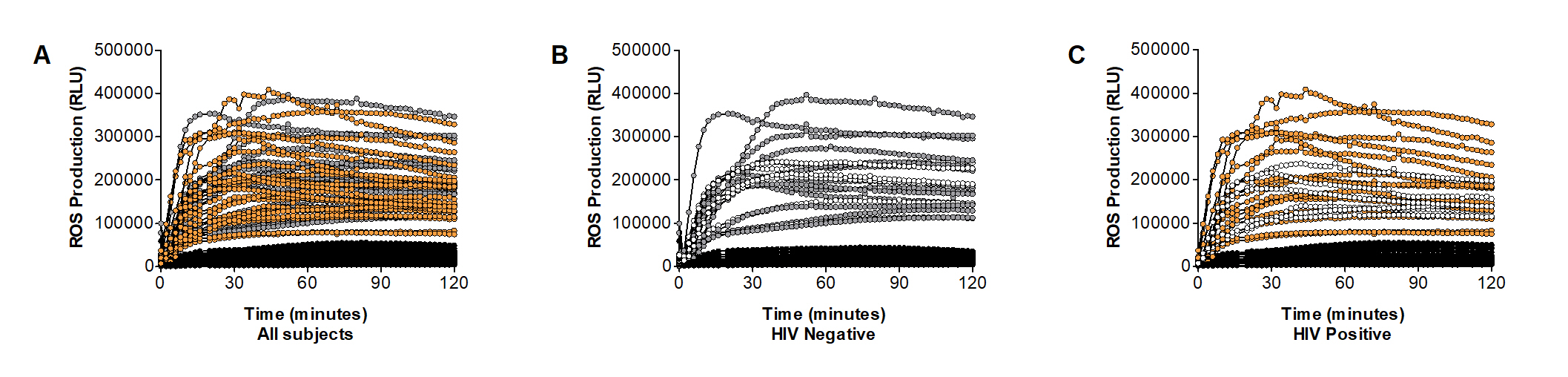

Supplement: Supplementary Figure 3 — Neutrophil Reactive Oxygen Species (ROS) production time course. (A) Neutrophil ROS production in all individuals as measured by chemi-luminescence (relative light units, RLU) every 2 min over a 120 min time interval. Black circles indicate unstimulated samples. Stimulated samples are indicated by gray (HIV negative) and orange (HIV positive) circles. (B) HIV negative individuals are further presented by DARC status where DARC-null individuals are represented as gray circles and DARC-positive individuals are represented as white circles. (C) HIV positive individuals are further represented by DARC status where DARC-null individuals are represented as orange circles and DARC-positive individuals are represented as white circles. Circles indicate data points for each participant joined by lines over the 120 min time interval. ROS, reactive oxygen species; RLU, Relative Light Units; DARC, Duffy Antigen Receptor for Chemokines. [file Image_3.JPEG]

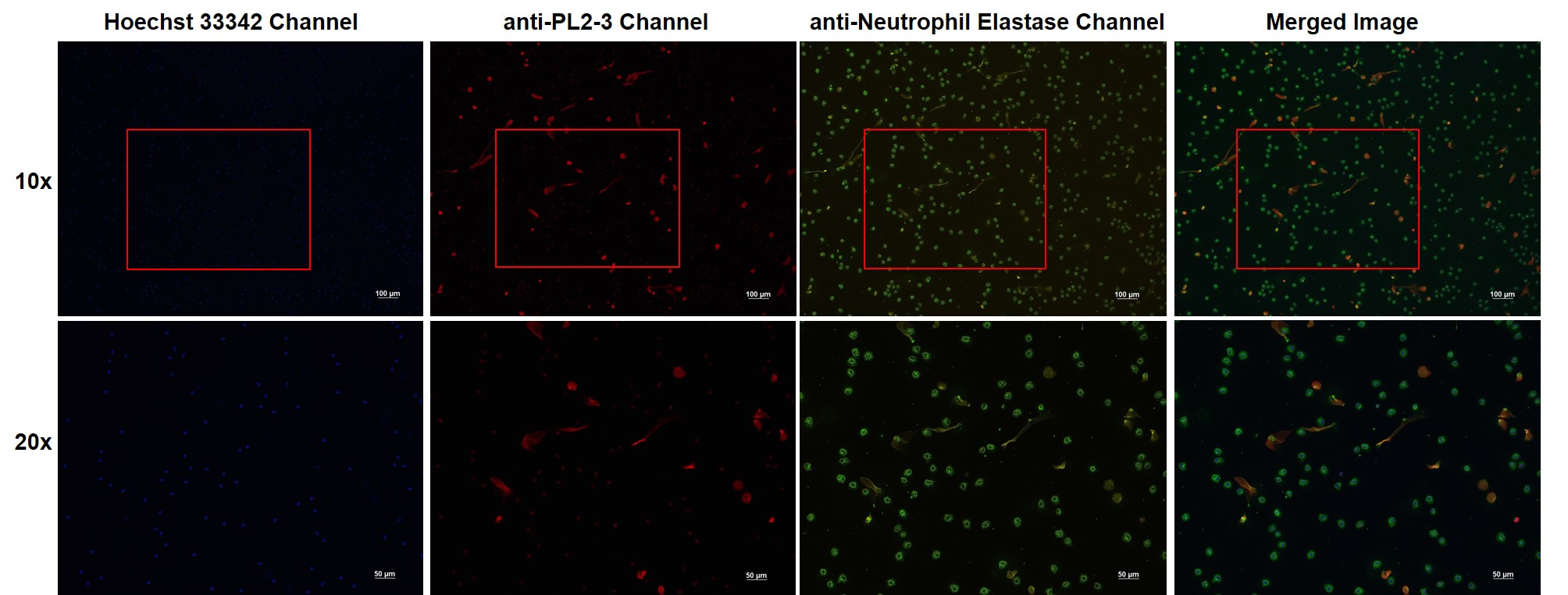

Supplement: Supplementary Figure 4 — Neutrophil Extracellular Trap Microscopy. Representative microscopy images showing Hoechst (blue), anti-PL2-3 (red), and anti-neutrophil elastase (green) staining staining at 10x (top panel) and 20x (bottom panel) magnification after 60 min post PMA activation. Overlaying of the three channels created the merged image. Red boxes indicate the region shown in the 20x magnification panel. [file Image_4.JPEG]
